# Does DNA replication direct locus-specific recombination during host immune evasion by antigenic variation in the African trypanosome?

**DOI:** 10.1007/s00294-016-0662-7

**Published:** 2016-11-07

**Authors:** Rebecca Devlin, Catarina A. Marques, Richard McCulloch

**Affiliations:** 1 The Wellcome Trust Centre for Molecular Parasitology, Institute of Infection, Immunity and Inflammation, University of Glasgow, Sir Graeme Davis Building, 120 University Place, Glasgow, G12 8TA UK; 2 Division of Biological Chemistry and Drug Discovery, College of Life Sciences, University of Dundee, Dundee, UK

**Keywords:** Antigenic variation, Trypanosome, DNA repair, DNA replication, Variant surface glycoprotein

## Abstract

All pathogens must survive host immune attack and, amongst the survival strategies that have evolved, antigenic variation is a particularly widespread reaction to thwart adaptive immunity. Though the reactions that underlie antigenic variation are highly varied, recombination by gene conversion is a widespread approach to immune survival in bacterial and eukaryotic pathogens. In the African trypanosome, antigenic variation involves gene conversion-catalysed movement of a huge number of variant surface glycoprotein (VSG) genes into a few telomeric sites for VSG expression, amongst which only a single site is actively transcribed at one time. Genetic evidence indicates VSG gene conversion has co-opted the general genome maintenance reaction of homologous recombination, aligning the reaction strategy with targeted rearrangements found in many organisms. What is less clear is how gene conversion might be initiated within the locality of the VSG expression sites. Here, we discuss three emerging models for VSG switching initiation and ask how these compare with processes for adaptive genome change found in other organisms.

## Introduction

The genome is the crucible of life, with the genetic content acting as an information source for the generation and functioning of an organism (Hutchison et al. 2016) and nucleic acid structure directing genome copying during organism reproduction. The DNA genomes of all cellular organisms are subject to a wide range of damage, which results in diverse lesions that include modified or lost bases, base mismatches, aberrant intra- or inter-strand chemical bonds and single- or double-strand breaks (DSBs) in the phosphodiester backbone. A multitude of damage repair and tolerance pathways have evolved to protect genome integrity and allow continued replication in the face of this range of lesions. Conservation of these genome maintenance pathways across the three domains of life is apparent (Aravind et al. 1999; Eisen and Hanawalt 1999), though alterations and divergence are found, at least in part due to tailoring to suit the ecological requirements of specific organisms and cells (Omelchenko et al. 2005). Within the eukarya, protozoa have provided a wealth of experimental data illustrating functional diversification in genome maintenance (Genois et al. 2014; Lee et al. 2014; Machado et al. 2014; Passos-Silva et al. 2010).

The primary function of genome maintenance is to protect the fidelity of the genome’s structure and sequence content, since defective or altered repair responses can be lethal or lead to diseased states in metazoans. However, genomes are rarely invariant over multiple generations and small or large changes in composition, either generated by inaccurate replication or repair, or due to the action of genome parasites such as transposable elements, can be beneficial and be retained. In fact, genome variation in some organisms occurs at elevated levels, even within a single generation. Such variation can be genome-wide, including developmental chromosome fragmentation in ciliates such as *Tetrahymena* (Yao et al. 2014), and stochastic chromosome ploidy and gene copy number variation in *Leishmania* (Sterkers et al. 2014; Ubeda et al. 2014). In other settings, lesions are deliberately generated within specific regions of the genome to elicit repair and generate targeted change. This more localised change can be highly specific, such as alternating between two mating types in yeast (Lee and Haber 2015). However, broader variation in gene content is also seen, including during maturation of the immune system in mammalian development, where lesions throughout antigen receptor loci generate rearrangements that lead to the myriad of receptors and antibodies expressed by mature T and B cells (Roth 2014). A combination of these extremes, generating pronounced diversity from a localised site, is seen in many organisms that must survive hostile environments, with a widespread example being changes in surface antigens to thwart host adaptive immunity: a process termed antigenic variation (Deitsch et al. 2009).

In common with the range of genotoxic lesions organisms encounter and the range of genome maintenance pathways available, multiple lesion types initiate targeted sequence change and multiple repair pathways elicit the change. For instance, *Saccharomyces cerevisiae* and *Schizosaccharomyces pombe* both use homologous recombination (HR) to switch mating type, but repair is initiated by locus-specific DSB formation by the HO endonuclease in the former (Lee and Haber 2015) and by induced replication stalling in the latter (Klar et al. 2014). Even more remarkably, the two different forms of antigen receptor-associated rearrangements that occur in mammals, which both exploit non-homologous end-joining (NHEJ) repair, use different routes for initiation: mature T cell receptor and immunoglobulin genes are formed after DSBs are generated by RAG transposase-related enzymes (Roth 2014), whereas immunoglobulin class switching relies on breaks that arise from transcription-linked base modification (Hwang et al. 2015). Extensive evidence indicates that gene conversion-based HR is a widespread route for antigenic variation in bacterial and eukaryotic pathogens, and the strategies used to initiate such change are now being unravelled (Morrison et al. 2009; Palmer and Brayton 2007; Vink et al. 2012). As outlined below, despite remarkable parallels between HR-catalysed antigenic variation in *Trypanosoma brucei* (a eukaryote) and *Neisseria* (a bacteria), a number of models have been proposed to account for initiation in the former, none of which appear comparable with the approach adopted by the latter.

## Homologous recombination catalyses antigenic variation in *T. brucei*

The core features of antigenic variation in *T. brucei* have been understood for many years (Matthews et al. 2015; McCulloch et al. 2015). Survival of the parasite in the vertebrate host requires a dense, protective coat composed of Variant Surface Glycoprotein (VSG)(Manna et al. 2014; Schwede et al. 2015), which is expressed by RNA Polymerase I from a telomeric expression site (ES) (Glover et al. 2013b; Gunzl et al. 2015). Multiple ES are found at the ends of the *T. brucei* chromosomes and, in each, the *VSG* gene is found in close proximity to the telomeric repeats (Ginger et al. 2002; Hertz-Fowler et al. 2008). Distinct ES are used when *T. brucei* resides in the salivary glands of the tsetse vector and in the mammal, where the ES are larger (around 10–60 kbp) due to co-transcription of the *VSG* with multiple expression site associated genes (*ESAG*s) (see Fig. 2). Invariably, the *VSG* and the *ESAG*s are separated by 70 bp repeats, sequence elements found upstream of virtually all of the thousands of *VSG*s in the *T. brucei* genome (Cross et al. 2014; Hovel-Miner et al. 2016; Marcello and Barry 2007). *VSG*s are only known to be expressed when present in the ES, and therefore, the silent *VSG* genes provide a huge archive of new VSG coats, with the genes located both in arrays in the subtelomeres of the 11 diploid chromosomes and in single copy in hundreds of smaller, aneuploid chromosomes. New VSG coats are expressed during an infection as host immunity against currently expressed VSGs eliminates some of the infecting trypanosome population (Hall et al. 2013; McCulloch and Field 2015; Mugnier et al. 2015). Individual *T. brucei* cells express only one VSG at a time, due to selective transcription of only one of the ~15 ES (Glover et al. 2013b; Gunzl et al. 2015). VSG switching is stochastic, pre-emptive of host immune VSG recognition and can occur either by coordinated silencing of the active ES and activation of a silent ES, or by recombination reactions that replace the ES *VSG* with a silent gene from anywhere in the archive. Why *T. brucei* uses, possibly uniquely (Morrison et al. 2009), both transcription- and recombination-based strategies for antigenic variation is unclear. Indeed, though a wide range of factors have been described that influence singular ES expression, the processes that trigger and execute a transcriptional switch remain unclear (Batram et al. 2014; Cestari and Stuart 2015; Figueiredo et al. 2008; Glover et al. 2013b, 2016; Gunzl et al. 2015). In contrast, catalysis of VSG switching by recombination is becoming clearer, and work is beginning to test how this directed genetic change is initiated.

**Fig. 1:**
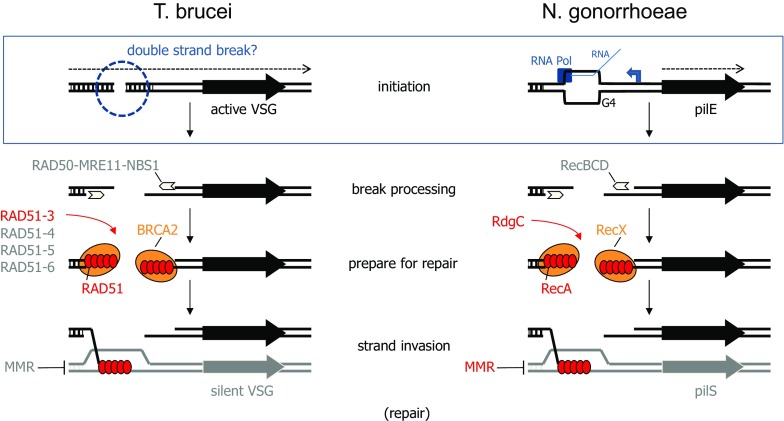
A comparison of the early steps in antigenic variation by gene conversion in *Trypanosoma brucei* and *Neisseria gonorrhoeae.* The involvement of factors that have been tested for a role in catalysis of the early steps of antigenic variation by homologous recombination (HR) in *T. brucei* (*left*) and *N. gonorrhoea* (*right*) are shown; factors shown in colour have been found to act, while those for whom no evidence of a role in antigen switching has been found are shown in grey. HR repair steps after DNA strand exchange are not shown, and the functions of most factors are discussed in the text. Mismatch repair (MMR) has been shown to suppress pilin switching in *N. gonorrhoea*, but not to suppress *T. brucei* VSG switching. For both pathogens the actively transcribed surface antigen gene (active *VSG* and *pilE*) is shown as a *black arrow*, and a silent copy of the genes that provide a substrate for HR repair are shown as *grey arrows* (silent *VSG*, *pilS*). Hatched boxes denote upstream regions of sequence homology used during HR of the antigen genes. For convenience, the action of the HR factors in both pathogens is shown as acting on a DNA double-strand break, but the role of such a lesion in initiating antigen switching in both cases is open to question. The *insert box* provides details of the current model for pilin switch initiation, which involves formation of a G quadruplex (G4) structure after passage of RNA polymerase (RNA Pol) transcription from a promoter (*dotted arrow*) that is oriented away from the transcribed *pilE* locus (*dotted arrow*). *VSG* transcription (*dotted arrow*) emanates from a distant promoter (not shown) and the initiating events in VSG switching are less clear, but may involve DSBs formed upstream of the antigen gene

Though a number of reactions have been described for activation of silent *VSG*s by recombination (McCulloch et al. 2015), the most common pathway is gene conversion, where a copy is generated of a silent *VSG* and replaces the *VSG* in the ES (Fig. 1). However, a complication is that *VSG* gene conversion could actually take place via two pathways: copying of a complete, functional *VSG*; and assembly of a novel ‘mosaic’ *VSG *(Barbet and Kamper, 1993) by segmental gene conversion from multiple silent *VSG*s. Only the latter reaction is able to generate a new VSG coat from *VSG* pseudogenes, which comprise the majority of the silent archive (Berriman et al. 2005; Cross et al. 2014; Marcello and Barry 2007). Segmental *VSG* conversion predominates during antigenic variation as *T. brucei* infections progress and has the capacity to generate VSG coat diversity beyond the number of *VSG* genes in the archive (Hall et al. 2013; Marcello and Barry 2007; McCulloch and Field 2015; Mugnier et al. 2015; Roth et al. 1989). Unfortunately, despite the importance of segmental *VSG* conversion, the genetic studies conducted to date to examine the machinery and sequences that act in VSG switching have been limited to intact *VSG*s, which are more readily activated. Thus, we cannot yet say, if segmental *VSG* conversion deviates (in machinery, mechanism and location) from the findings discussed below for intact *VSG* recombination.

Genetic analyses conducted independently have revealed striking similarities in the use of HR to catalyse antigenic variation in *T. brucei* and in *Neisseria gonorrhoeae* (where immune evasion occurs by gene conversion of silent, non-functional *pilS* genes into a *pilE* expression locus) (Obergfell and Seifert 2015) (Fig. 1). In both pathogens, ablation of the central enzyme of HR-Rad51 (McCulloch and Barry 1999) or RecA (Koomey et al. 1987), respectively—significantly impairs antigenic variation. Indeed, mutation of genes encoding proteins that aid the HR recombinases have a similar outcome. Though not homologues, BRCA2 and RecX both function during HR by modulating Rad51/RecA nucleoprotein filament formation; loss of BRCA2 in *T. brucei* (Hartley and McCulloch 2008; Trenaman et al. 2013) and RecX in *N. gonorrhoeae* impairs antigen gene switching (Stohl and Seifert 2001). Similarly, mutation of at least one RAD51 paralogue in *T. brucei* impedes VSG switching (Dobson et al. 2011; Proudfoot and McCulloch 2005), while loss of RdgC has the same effect on *N. gonorrhoeae* pilin switching (Mehr et al. 2000); again, despite bacterial RdgC and eukaryotic Rad51 paralogues not being clearly homologous, both act to modulate Rad51- (Gaines et al. 2015; Taylor et al. 2015) and RecA-mediated (Briggs et al. 2010) strand exchange activity. These data suggest both pathogens have co-opted the general repair strategy of HR to catalyse locus-specific, frequent gene recombination. However, though Fig. 1 depicts the HR factors as acting to repair a DSB, whether such a lesion directs antigenic variation in the two pathogens remains unclear, in part due to what we are learning about how these processes are initiated.

## How is *T. brucei* VSG switching initiated?

Understanding the lesion(s) that triggers VSG switching in *T. brucei* is important: catalysis of antigenic variation by the well conserved repair pathway of HR makes it an unattractive target to block the process for disease therapy, whereas initiation may yet reveal a lineage-specific reaction and machinery that could be a drug target. Such putative interventions need not necessarily act on the initiating process, but could target how initiating lesions are recognised and signalled to execute repair. In this regard, further genetic overlap between *T. brucei* and *N. gonorrhoeae* antigen gene switching may be revealing. A key step in DSB repair by HR is end-processing to yield single stranded ends for Rad51 and RecA filament formation. Eukaryotes and bacteria possess distinct protein complexes that provide the major contribution to DSB resection during HR (Symington 2014): Mre11-Rad50-Nbs1/Xrs2 (MRN) and RecBCD, respectively. In both *T. brucei* (Robinson et al. 2002; Tan et al. 2002) and *N. gonorrhoeae* (Helm and Seifert 2009) mutation of components of these complexes results in HR repair deficiency, but in neither case is antigen gene switching rate affected, arguing that in both systems of immune evasion the expected primary DSB-processing complex is not recruited to the initiating lesion(s) (Fig. 1). An explanation for this finding in *N. gonorrhoeae* lies in recent data showing pilin switch initiation is remarkably complex and does not involve the direct formation of a DSB (Fig. 1). Mutagenesis revealed that a guanine quadruplex (G4) forming sequence (Cahoon and Seifert 2009) and a promoter driving expression of a short RNA (transcribed in the opposite direction to the *pilE* locus) (Cahoon and Seifert 2013) are needed for pilin switching. As DNA nicks can be detected in the G4 motif, which recruits RecA (Kuryavyi et al. 2012) and can be unwound by RecQ helicase activity (Cahoon et al. 2013), a model for initiation is suggested (Obergfell and Seifert 2015) in which transcription across the G4 sequence results in an RNA-DNA hybrid on the C-rich strand, which allows the G4 structure to form and leads to nicks on the opposite strand (by unknown means). Initiation by DNA nicks, which could be processed to yield single strand gaps, would explain the lack of involvement of RecBCD and the need for the RecF pathway in pilin switching (Helm and Seifert 2010).

To date, no single model for initiation of *T. brucei* VSG switching has gained prominence. A simple copy of the strategy used by *N. gonorrhoeae* seems very unlikely, since multigenic ES transcription to generate *VSG* mRNA appears very different from *pilE* transcription (Fig. 1). In addition, the *VSG*-associated 70 bp repeats appear to have different characteristics to G4-forming pilin sequences, with the available data suggesting that a triplet repeat component of the 70 bp repeats causes a propensity to become non-H bonded (Ohshima et al. 1996). Indeed, as attractive as the 70 bp repeats are as an initiation feature for VSG switching, the available evidence on their importance is strikingly contradictory. Two studies have reported that deletion of the 70 bp repeats from the active ES does not to affect VSG switching frequency (Boothroyd et al. 2009; McCulloch et al. 1997), though does impede elevated *VSG* recombination after deliberately introducing a DSB (below). In contrast, a recent study has suggested that loss of the 70 bp repeats, with or without the generation of a DSB in the active ES, dramatically increases VSG switching (Hovel-Miner et al. 2016). Moreover, though DNA breaks around the 70 bp repeats in the active ES has been reported (Boothroyd et al. 2009), further studies have not detected such a strict localisation to these sequence elements or limitation to the active ES (Glover et al. 2013a; Jehi et al. 2014).

**Fig. 2:**
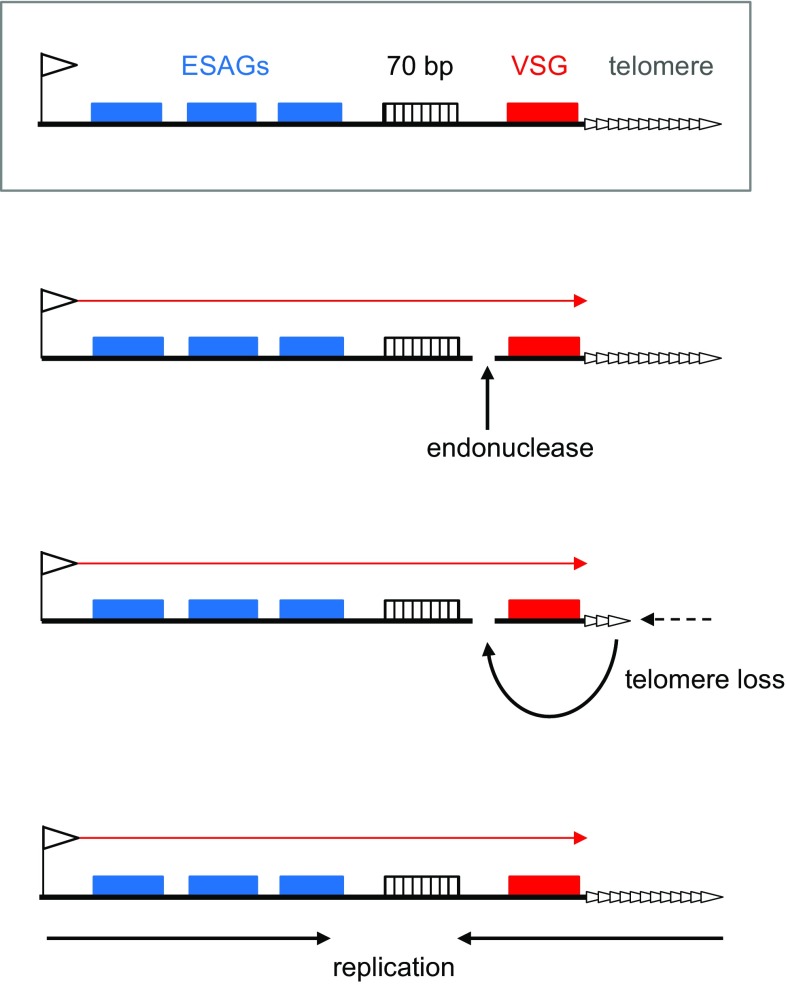
Three models for initiation of antigenic variation in *Trypanosoma brucei*. The *upper, boxed diagram* is a schematic of a bloodstream VSG expression site (ES, not to scale), detailing key features (*left to right*): the promoter (flag), a number of expression site associated genes (*ESAG*s; *blue boxes*), 70 bp repeats (*hatched box*), the *VSG* gene (*red box*) and the telomere repeats (*white arrows*). Transcription (*red arrow*) is detailed in the three models below, which compare proposed strategies for initiation of VSG switching that involves recombination-based removal of the *VSG* from the active ES: the targeted action of an endonuclease generates a double-strand break (DSB; shown for convenience between the 70 bp repeats and *VSG*); loss of telomere repeats transmits a break (shown as a DSB) into the ES; and specific, early replication (*black arrow*) of the actively transcribed ES (it is unknown if replication is co-directional with transcription, or in opposition)

Currently, three models for VSG switch initiation have been proposed (Fig. 2). The direct formation of a DSB through the action of an endonuclease in the ES has long been discussed (Barry 1997; Borst et al. 1996). Boothroyd et al. (2009) demonstrated this is feasible, since targeting the yeast I-SceI endonuclease to a recognition sequence in the active ES elevates VSG switching through gene conversion. Indeed, and as extended by a further study (Glover et al. 2013a), location of the I-SceI-generated DSB in the ES is important: only a DSB adjacent to the 70 bp repeats in the active ES caused *VSG* gene conversion; a DSB in an inactive ES or adjacent to the active ES promoter did not induce VSG switching, and a DSB downstream of the *VSG* and adjacent to the telomere repeats caused a modest increase in VSG switching, but through ES deletion not *VSG* gene conversion. However, whether or not *T. brucei* possesses an endonuclease whose role in VSG switching is modelled by I-SceI is unknown. One problem with this model is that an endonuclease-generated DSB appears incompatible with several genetic analyses. As noted above, endonuclease-generated DSBs in eukaryotes are first recognised and processed by MRN (Symington 2014), which appears not to contribute to VSG switching (Robinson et al. 2002). More extensive end resection is then catalysed by a complex composed of a RecQ helicase (Sgs1 in yeast, BLM in mammals), Top3 (a topoisomerase), Rmi1 and Dna2 (an endonuclease). Mutation of RECCQ2, the likely helicase component of this RTR complex in *T. brucei*, impairs repair of I-SceI catalysed DSBs and the protein localises to induced damage along with RAD51, consistent with the same repair activity in the parasite (Devlin et al. 2016). Despite this function, mutation of RECQ2 causes increased, not decreased levels of VSG switching and a pronounced shift in the recombination to reciprocal exchanges between telomeres, phenotypes also seen in mutants of two other putative RTR complex components (Kim and Cross 2010, 2011) and consistent with downstream actions in controlling HR strand exchange (Haber 2015).

A second, somewhat overlapping model derives from the telomere-proximity of *VSG*s in all ES (Fig. 2). Here, telomere length is the key determinant, with VSG switching proposed to be initiated by ES breaks that derive from critically short telomeres (Dreesen et al. 2007; Hovel-Miner et al. 2012; Li 2015). This suggestion was prompted by studies revealing that, while all *T. brucei* telomeres grow with each cell division, growth of the active ES telomere is more rapid and fragmentation leads to greater telomere length heterogeneity at this site in the population (Pays et al. 1983). Support for the model comes from telomerase-deficient *T. brucei* mutants, which gradually lose telomere repeats and, when very short, VSG switching is increased (Hovel-Miner et al. 2012). In addition, loss of TIF2 (one component of the shelterin complex that binds chromosome ends) results in increased breaks within the ES and increased VSG switching (Jehi et al. 2014), an effect also seen after loss of a second shelterin component, TRF (though without detectably increased ES breaks) (Jehi et al. 2016). How breaks are transmitted from short or unprotected telomeres, and the form they take is unclear. In addition, the significance of the above observations in telomerase- and shelterin-proficient cells needs to be addressed, including potentially how the effects on VSG switching might relate to known telomere stabilisation reactions that take effect on short telomeres (Dreesen et al. 2005).

A third model proposes a quite distinct strategy for VSG switch initiation. Recent work has sought to map the dynamics of nuclear DNA replication in *T. brucei*, revealing three things (Devlin et al. 2016; Tiengwe et al. 2012). First, the location and timing of replication initiation is highly invariant in the core *T. brucei* genome (excluding the *VSG* archive). Second, most of the subtelomeres, including all the silent ES, are late replicating. Third, the active ES, alone among all the ES, replicates early in mammal-infective cells, and this differential timing is dependent on transcription status, since the site becomes late-replicating in tsetse-infective cells (when all ES are silent) and when silenced by blocking transcription elongation. This precise association between replication timing and singular ES activity suggests that early replication through the ES might be the driver for VSG switching initiation, explaining the likely focus on the active ES (Fig. 2). How, in detail, this might occur is unknown, but the increased levels of VSG switching and altered route of recombination in RECQ2 (Devlin et al. 2016), TOP3a (Kim and Cross 2010) and RMI1 (Kim and Cross 2011) mutants may be consistent with the mode of action of the RTR complex on stalled replication forks (Hickson and Mankouri 2011). Indeed, clashes between replication and transcription are pronounced sources of instability in all organisms (Bermejo et al. 2012). Nonetheless, tests of the proposed association between replication timing and VSG switching are needed, including mapping replication direction through the ES (and the associated subtelomere), and asking if replication stalling occurs in the ES and leads to detectable switch intermediates. Determining what machinery recognises and signals any putative clashes is also needed, as is unravelling the potential significance and specificity of telomere binding by the replication initiator ORC1/CDC6 (Benmerzouga et al. 2013). Intriguingly, a homologue of *T. brucei* ORC1/CDC6 has also been shown to localise to subtelomeres in *Plasmodium falciparum*, where antigenic variation is also orchestrated (Mancio-Silva et al. 2008).

## What does telomeric VSG expression reveal about replication timing in eukaryotes?

Replication in all cellular organisms normally initiates from genomic sites called origins. In bacteria and some archaea, only a single origin is found that is activated through binding of replication initiators in every cell division. In contrast, the chromosomes of most eukaryotes and some archaea possess multiple origins, which show greater flexibility in usage. In most characterised eukaryotes and some archaea (Yang et al. 2015) larger numbers of genomic sites are defined by initiator binding that are activated during each cell cycle, and some initiator sites act as dormant origins that can be activated during replication stress (Alver et al. 2014; Mechali 2010). In addition, the frequency with which origins are activated in the population, or the timing of origin activation during S phase, is variable (Rhind and Gilbert 2013; Rivera-Mulia et al. 2015). Centromeres are notably early replicating sites, while telomeres are notably late-replicating and, at least in large metazoan genomes, clusters of early and late acting origins can define larger domains of replication timing. Replication timing has been associated with density of replication factor recruitment, transcription activity, chromatin status and with organisation of the chromosomes within the nucleus. Given the relatively small size of the genomes of *T. brucei* and the related kinetoplastid parasite *Leishmania*, and the organisation of the core into discrete, well understood multigene transcription units, replication profiling in a population most likely identifies origins (Marques et al. 2015; Tiengwe et al. 2012) and not replication timing domains (Lombrana et al. 2016; Rocha-Granados and Klingbeil 2016), meaning such analysis is more comparable with yeast (McGuffee et al. 2013; Muller et al. 2014) than with metazoans (Petryk et al. 2016). If so, what features in *T. brucei* dictate origin ‘strength’ appear unconnected with transcription activity, since variable strength origins, as well as origin-inactive ORC1/CDC6 sites, bind to transcription starts sites of the multigene units (Tiengwe et al. 2012), for which there is no evidence of differential transcription. Indeed, in this context, *Leishmania* appears strikingly discordant from *T. brucei*, with only a single discrete, highly active origin identified per chromosome using the same replication mapping strategy (Marques et al. 2015). The mechanistic basis of this replication dichotomy in the highly syntenic genomes of two kinetoplastids remains the subject of debate (Lombrana et al. 2016; Marques et al. 2015). Nonetheless, if transcription level does not dictate replication timing throughout the *T. brucei* genome, then the precise correlation between VSG ES activity and early or late replication means either other genome functions dictate timing (e.g. chromatin or subnuclear location), or this single genome loci is an exception to the rules. Irrespective of the explanation, the diverged mode of gene expression, allied to the precision of replication timing and ES activity linkage, suggests *T. brucei* and related kinetoplastids can provide insight into how replication dynamics relate to genome organisation and expression in all eukaryotes.
